# Characterization and Comparative Analysis of Two Rheum Complete Chloroplast Genomes

**DOI:** 10.1155/2020/6490164

**Published:** 2020-06-19

**Authors:** Kaihui Zhao, Lianqiang Li, Yazhou Lu, Junbo Yang, Zhirong Zhang, Fangyu Zhao, Hong Quan, Xiaojing Ma, Zhihua Liao, Xiaozhong Lan

**Affiliations:** ^1^TAAHC-SWU Medicinal Plant Joint R&D Center, Tibetan Collaborative Innovation Center of Agricultural and Animal Husbandry Resources, Food Science College, Tibet Agriculture & Animal Husbandry University, Nyingchi, Tibet 860000, China; ^2^Germplasm Bank of Wild Species, Kunming Institute of Botany, Chinese Academy of Sciences, Kunming, Yunnan 650201, China; ^3^Key Laboratory of Forest Ecology in Tibet Plateau (Tibet Agricultural & Animal Husbandry University), Ministry of Education, Nyingchi, Tibet 860000, China; ^4^State Key Laboratory of Dao-di Herbs, National Resource Center for Chinese Materia Medica, China Academy of Chinese Medical Sciences, Beijing 100700, China; ^5^Key Laboratory of Eco-environments in the Three Gorges Reservoir Region, Ministry of Education, Chongqing Engineering and Technology Research Center for Sweetpotato, School of Life Sciences, Southwest University, Chongqing 400715, China

## Abstract

*Rheum* species present a significant economic value. Traditional Chinese medicine rhubarb is an important medicinal material in China. It has a long history of use, with a record of use as early as two thousand years ago. Here, we determined the complete chloroplast genome sequences of *Rheum nobile* and *Rheum acuminatum* and comprehensively compared them to two other available *Rheum* cp genomes at the genome scale. The results revealed cp genomes ranging in size from 159,051 to 161,707 bp with a similar typical quadripartite and circular structure. The genome organization, gene numbers, gene order, and GC contents of these four *Rheum* cp genomes were similar to those of many angiosperm cp genomes. Repeats and microsatellites were detected in the *R. nobile* and *R. acuminatum* cp genomes. The Mauve alignment revealed that there were no rearrangements in the cp genomes of the four *Rheum* species. Thirteen mutational hotspots for genome divergence were identified, which could be utilized as potential markers for phylogenetic studies and the identification of *Rheum* species. The phylogenetic relationships of the four species showed that the members of Rheum cluster into a single clade, indicating their close relationships. Our study provides valuable information for the taxonomic, phylogenetic, and evolutionary analysis of *Rheum*.

## 1. Introduction

The genus Rheum, belonging to the family Polygonaceae, is mainly distributed in the temperate and subtropical alpine regions of Asia [1]. *Rheum* has a long history of medicinal use in China [1]. The output and quality of *Rheum* used for Chinese medicine rank first in the world, especially in Tibetan medicine, which uses *Rheum* plants particularly effectively. Extracts of *Rheum* have various pharmacological effects, such as purgation, antibacterial, anti-inflammatory, antiviral, and antitumor effects [2–5]. People in Tibet and Sichuan and Qinghai Provinces in China not only use the roots of rhubarb as medicinal materials but also use the petioles and young stems and leaves of the aerial parts of *Rheum* to quench their thirst and treat the symptoms of the eyes [2]. Nevertheless, with the increasing market demand for *Rheum*, the phenomenon of indiscriminate digging up of *Rheum* is increasing, wild *Rheum* resources have been severely damaged, and the ecological environment of *Rheum* plants has also been damaged to varying degrees.

At present, the research on *Rheum* plants is mainly focused on the investigation of their resource distribution [1, 6], chemical composition [7–11], pharmacological effects [12–15], and side effects [16]. Because *Rheum* comprises highly phenotypically plastic Chinese herbal medicine species, there are significant differences in the quality and efficacy of different species or varieties of the same origin [2]. The comparative study of the genetic relationships among various species and the systemic evolution of *Rheum* promote its scientific use in medicine. Chloroplasts (cp) play important roles in photosynthesis, carbon fixation, and starch and fatty acid biosynthesis [17]. A complete cp genome is a valuable source of information for studying plant taxonomy and performing phylogenetic reconstruction and historical biogeographic inferences. Some scholars believe that the entire sequence of the cp genome can be used as an ultrabarcoding, and the concept of a super barcode has been proposed [18]. Subsequent research has shown that the use of cp genome sequences can improve the ability to identify allied groups [19, 20].

In the present study, we obtained the cp genomes of *Rheum nobile* Hook. f. et Thoms. and *Rheum acuminatum* Baill. through Illumina sequencing and characterized long repeats and simple sequence repeats in these genomes. We analyzed and compared the cp genomes of *R. nobile* and *R. acuminatum* and the cp genomes of other members of *Rheum*. Combined with previously reported cp genome sequences downloaded from the National Center for Biotechnology Information (NCBI) database for other members of *Rheum* [21], these complete cp genomes will increase our understanding of the phylogenetic relationship of *Rheum* species. Our studies could provide basic data for medicinal species conservation and molecular phylogenetic research in *Rheum*.

## 2. Results and Discussion

### 2.1. Genome Organization and Features

**Figure 1:**
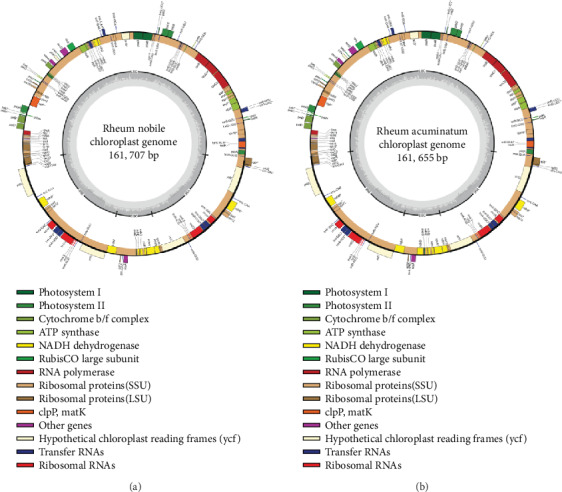
The complete cp genome map of *R. nobile* (a) and *R. acuminatum* (b). Genes in the circle are transcribed clockwise, while the rest are transcribed counterclockwise. Dark gray shading in the inner circle indicates the GC content.

209,899 and 80,807 clean reads were mapped to the relative species (*R. palmatum* NC027728), after screening these clean reads by aligning them to *R. palmatum* cp genome, on average reaching over 100x coverage of the cp genome. The cp genomes of *R. nobile* and *R. acuminatum* exhibit a typical quadripartite structure with a conserved genome arrangement and are similar to those of *R. wittrockii* and *R. palmatum*. The cp genomes of *R. nobile* and *R. acuminatum* are 161,707 and 161,655 bp in size, respectively, including two inverted repeats (IRs) separated by a large single copy (LSC) region and a small single copy (SSC) region (Figure 1). Each of the cp genomes encode 131 genes, including 86 protein-coding genes, 37 transfer RNA genes, and 8 ribosomal RNA genes. Among these unique genes, fifteen genes have one single intron, and two (*ycf3* and *clpP*) contain two introns (Table S1). The length of the SSC region is 12,839 bp and 12,806 bp in *R. nobile* and *R. acuminatum*, respectively, and that of the LSC region is 86,778 bp and 86,909 bp, respectively. The GC content of these *Rheum* cp genomes is ~37% (Table 1); the GC content of the IR regions is ~41%, and those of the LSC and SSC regions are ~35% and 32% (Table 1), respectively. The GC content of the IR region is clearly higher than that of the other regions (LSC, SSC); thus, our findings are in concordance with other research results [22, 23].

### 2.2. Repeat Structure and Simple Sequence Repeat Analyses

**Figure 2:**
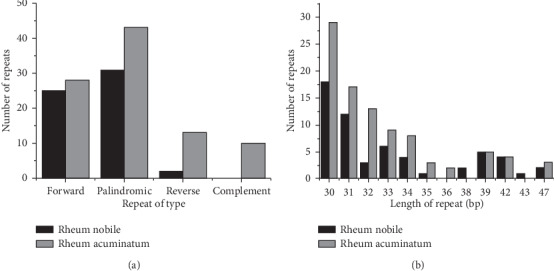
Analysis of repeated sequences in the *R. nobile* and *R. acuminatum* cp genomes.

Repeat sequences contain a large amount of genetic information and may promote the rearrangement of the cp genome and increase the genetic diversity of a population [24]. Repeat sequences in two cp genomes were analyzed using Vmatch, with the criterion of a copy size of 30 bp or longer. A total of 58 and 93 pairs of repeats were identified in the *R. nobile* and *R. acuminatum* cp genomes (Figure 2(a)). *R. nobile* contained 25 forward repeats, 31 palindromic repeats, 2 reverse repeats, and 0 complement repeats and *R. acuminatum* contained 28, 42, 13, and 10 repeats, respectively, with repeat lengths ranging from 30 to 47 bp (Figure 2(b)).

**Figure 3:**
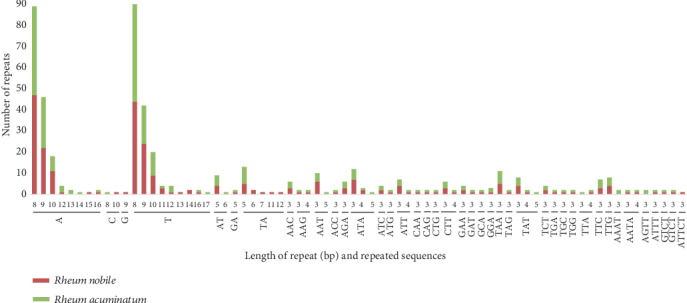
Analysis of simple sequence repeats in the *R. nobile* and *R. acuminatum* cp genomes.

Simple sequence repeats (SSRs) are important molecular markers for species authentication and analysis of plant population genomics and evolutionary history [25, 26]. Here, a total of 244 and 243 SSRs were detected in the cp genomes of *R. nobile* and *R. acuminatum* (Figure 3), respectively. The majority of the 143 SSRs in the *R. nobile* cp genome are located in the LSC region (58.60%), while 72 are located in the two IR regions (29.50%), and 29 are located in the SSC region (11.90%), which was in accordance with previous research results [27]. The numbers and distribution of all SSR types were similar and conserved in the two cp genomes. Among these SSRs, mononucleotide repeats accounted for the highest proportion (Figure 3) of 66.39% in *R. nobile* and 69.96% in *R. acuminatum.* Only a minor fraction consisted of dinucleotide, trinucleotide, and tetranucleotide repeat motifs. SSRs were mainly located in IGS (42.80%) and CDS (41.56%) regions and were also detected in introns (15.64%), such as those of *matK*, *atpF*, *rpoC2*, *rpoC2*, *clpP*, *rpl2*, *ycf2*, *ycf1*, *ndhF*, and *ndhD*. These SSRs will highly beneficial for the development of useful molecular markers for the assessment of genetic diversity and population structure among the species of *Rheum* in future studies.

### 2.3. Comparison of the Basic Characteristics of the Chloroplast Genome in Four *Rheum* Species

We compared the cp genome characteristics of four *Rheum* species (Table 1). The cp genome length of the *Rheum* species ranged from 159,051 to 161,707 bp, with the shortest being found in *R. wittrockii* and the longest in *R. nobile*. The length of IRs in the *Rheum* species ranged from 30,651 to 31,045 bp, and the shortest length was again found in *R. wittrockii* and the longest in *R. nobile*. The SSC length ranged from 12,806 to 12,999 bp, with the longest being found in *R. palmatum* and the shortest in *R. acuminatum*. The numbers of protein-coding genes, tRNA genes, and rRNA genes in the genomes were 86, 37, and 8, respectively. The cp genomes of the four *Rheum* species exhibited the same sizes and numbers of genes, revealing that the cp genomes of *R. nobile* and *R. acuminatum* were similar to those of the other *Rheum* species; thus, slow evolution is a characteristic *Rheum* according to the comparison of the cp genomes of *R. nobile*, *R. palmatum*, *R. wittrockii*, and *R. acuminatum*.

**Figure 4:**
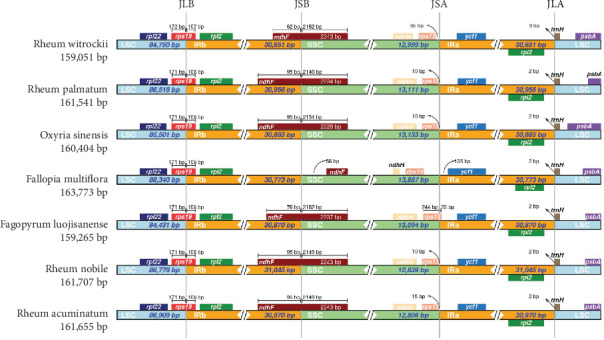
Comparison of the junctions of the large single copy (LSC), small single copy (SSC), and inverted repeat (IR) regions in the chloroplast genomes of Polygonaceae. JLB represents the of LSC/IRb junction, JSB represents the SSC/IRb junction, JSA represents the SSC/IRa junction, and JLA represents the LSC/IRa junction.

The contraction and expansion of the IR region determine the size of the cp genome [28]. The locations of SSC/IR and LSC/IR junctions are markers of cp genome evolution [29]. The IR boundaries were compared between *Rheum*, *Oxyria*, *Fallopia*, and *Fagopyrum* in Polygonaceae, including the above four *Rheum* species, *Oxyria sinensis*, *Fallopia multiflora*, and *Fagopyrum luojishanense* (Figure 4). Although the genomic structure and size were highly conserved among the Polygonaceae cp genomes, the IR boundary regions varied slightly. The IRa/SSC boundaries were located downstream of the *rps15* gene except in *F. luojishanense*, in which the *rps15* gene crossed over the IRa/SSC region. At the IRb/SSC junction, 62-95 bp of the *ndhF* gene was located within the IRb, while the rest was located in the SSC regions of the Polygonaceae members, except in *F. multiflora*, whose *ndhF* gene was fully located within the SSC region, 56 bp away from the SSC/IRb border. The analyses of IR boundaries showed that *Rheum* and *Oxyria* are closely related and that *Rheum* presents a closer genetic relationship to *Oxyria* than to *Fallopia* and *Fagopyrum*. The information generated from IR/SC junction regions and other variable regions from different Polygonaceae species would be useful for systematic and taxonomic analysis of other species of *Rheum* and other genera within the Polygonaceae.

**Figure 5:**
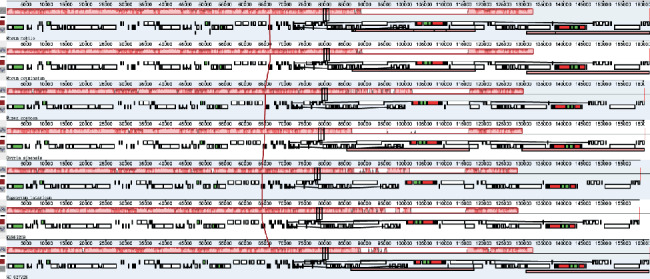
Gene arrangement map of Polygonaceae cp genomes and one reference species (*R. nobile*) aligned using Mauve software. Local collinear blocks within each alignment are represented as blocks of similar color connected with lines. Annotations of rRNA, protein-coding, and tRNA genes are shown in red, white, and green boxes, respectively.

The Mauve alignment for Polygonaceae species revealed that all the genomes formed locally collinear blocks (LCBs). According to the results regarding the collinear blocks of genes, including ribosomal RNA, tRNA, and protein-coding genes, the Polygonaceae genomes were relatively conserved, with no gene rearrangements (Figure 5). Some previous studies have also revealed the homology and an absence of gene rearrangements in genome organization; thus, our findings support their conclusions [24, 30].

**Figure 6:**
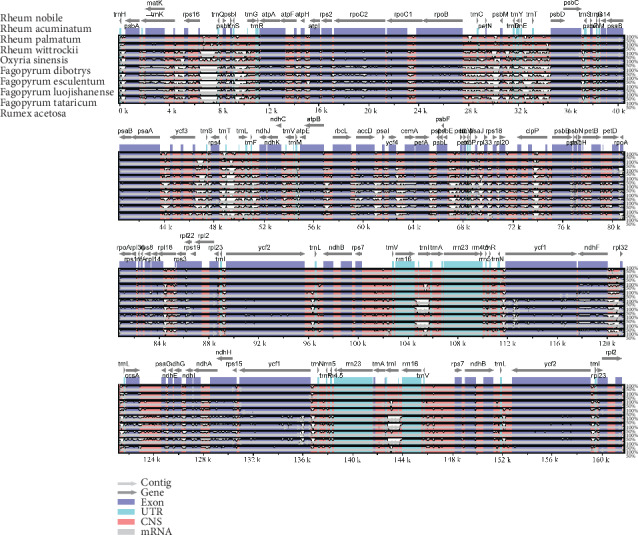
Sequence identity plot comparing ten cp genomes of Polygonaceae species with *R. nobile* as a reference by using mVISTA.

Interspecific comparisons between ten Polygonaceae species (Table S2) were conducted using mVISTA software with the annotated cp genome of *R. nobile* as a reference (Figure 6). Based on the overall sequence identity indicated by the peaks and valleys among all ten species of Polygonaceae, the results indicated that the LSC and SSC regions are more divergent than the two IR regions. The alignment revealed high sequence conservatism across the cp genomes of *Rheum*. Furthermore, the results showed that the coding regions are more conserved than the noncoding regions, as seen in other plants [27, 31]. According to the results of comparative analyses, some hotspot regions of genome divergence could be utilized as potential genetic markers for phylogenetic studies and the identification of Polygonaceae species. These highly divergent coding regions include the *rpoC2*, *matK*, *accD*, and *ndhF* regions, among others.

**Figure 7:**
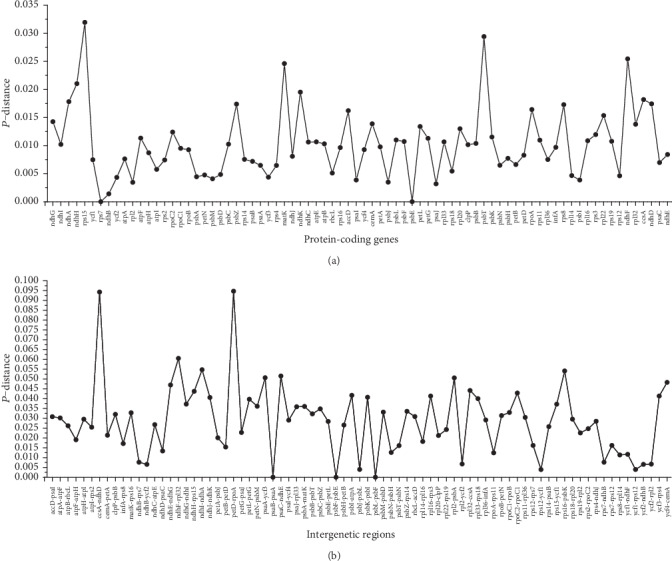
Nucleotide variability (PI) values were compared among four *Rheum* species. (a) *P*-distance values of protein-coding genes. (b) *P*-distance values of intergenic regions.

Four complete cp genomes of *Rheum* species, including those of *R. wittrockii* and *R. palmatum* and the newly assembled *R. nobile* and *R. acuminatum* cp genomes, were selected to analyze the DNA polymorphisms. The average value of nucleotide diversity (PI) across all 79 protein-coding genes was 0.00336, and five highly variable regions were identified based on a significantly higher PI value of >0.02. These highly divergent protein-coding genes included *rps15*, *psbT*, *ndhF*, *matK*, and *ndhH*, and the *rps15* gene was found to present the highest divergence value of 0.03194 (Figure 7(a)). The intergenic regions exhibited a relatively greater genetic distance compared to the protein-coding regions, ranging from 0 to 0.09469, with an average value of 0.02892. Eight intergenic regions were detected based on a significantly higher PI value of >0.05, including the *petD-rpoA*, *ccsA-ndhD*, *ndhF-rpl32*, *ndhI-ndhA*, *rps16-psbK*, *psaC-ndhE*, *psaA-ycf3*, and *rpl2-psbA* regions, and the *petD-rpoA* region was identified as showing the highest divergence value of 0.09469 (Figure 7(b)). These highly variable regions may be used as potential genetic markers for application in phylogenetic analysis and the identification of *Rheum* species.

### 2.4. Phylogenetic Analysis of the cp Genomes of *R. nobile* and *R. acuminatum*

**Figure 8:**
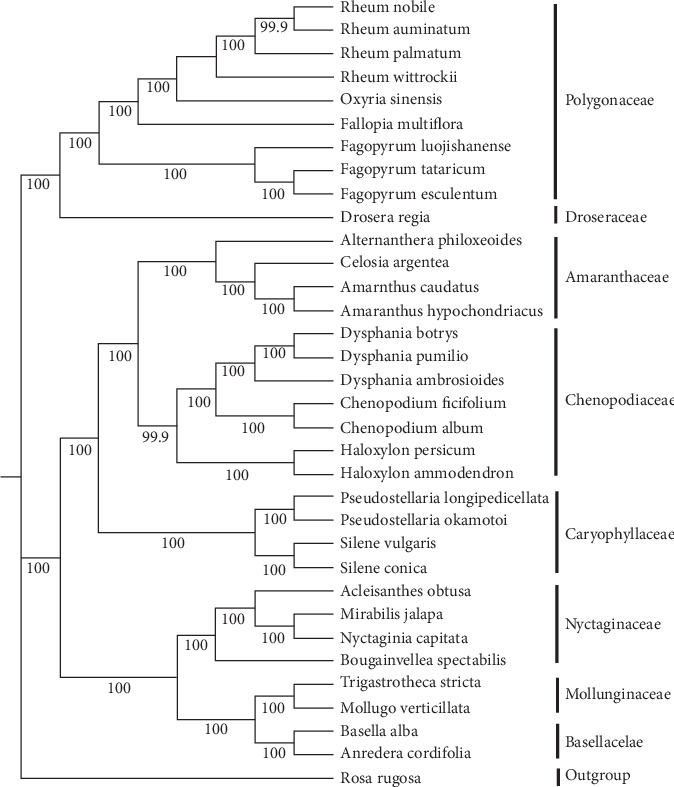
Phylogenetic tree reconstruction including 34 species based on all cp genomes. *Rosa rugosa* was used as the outgroup. The tree was generated using the ML method with 1000 bootstrap replicates. Numbers at the nodes indicate bootstrap values. The black triangle marks our newly assembled cp genome.

The chloroplast, mitochondrion, and nucleus provide independent genetic information in green plants [32]. Compared with other genomes, the chloroplast genome presents some unique advantages, such as a low nucleotide substitution rate, high accuracy and resolution, smaller size, and highly conserved genomic structure [32]. Therefore, complete cp genomes are frequently utilized in phylogenetic studies. To clarify the phylogenetic positions of *R. nobile* and *R. acuminatum* within the Caryophyllales, thirty-four species representing eight families from the order Caryophyllales were selected, and *Rosa rugosa* was selected as the outgroup. A phylogenetic tree was constructed using the maximum likelihood (ML) method (Figure 8). The result was consistent with the traditional plant morphological taxonomy. *R. nobile* and *R. acuminatum* clustered together to form a single clade, reflecting a closer relationship of these species. It is noteworthy that *Rheum* and *Oxyria* were grouped into a single clade with other closely related species of the Polygonaceae family. The results revealed that there are close relationships between *Rheum* and *Oxyria*, supporting the membership of both species in Subfam. Rumicoideae Damm. Sun et al. considered *Rheum* formed a monophyletic lineage sister to the genus Oxyria based on eight cp DNA fragments [33]. The phylogenetic relationship between the Polygonaceae and Droseraceae families was strongly supported by high bootstrap values, which is consistent with the APG IV system of Caryophyllales plant classification [34]. Droseraceae originally belonged to Sarraceniales [35], and our research supports the classification of Droseraceae (which shows a close relationship with Polygonaceae) into Caryophyllales. In the study by Su et al., Polygonaceae and Droseraceae also constitute a noncore Caryophyllales group [36].

**Figure 9:**
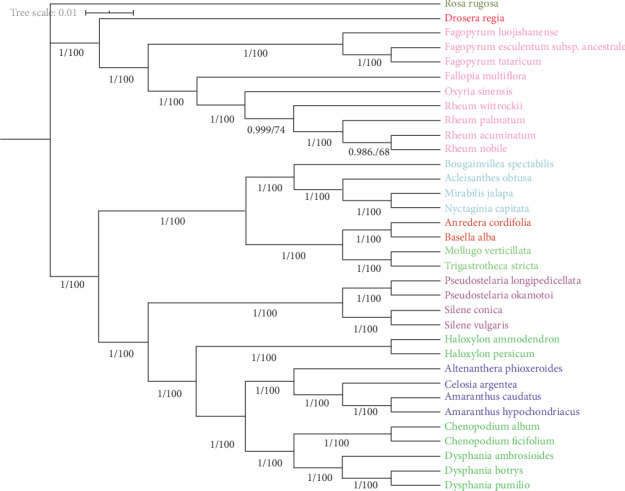
Phylogenetic tree reconstruction based on 53 protein-coding genes of 34 species cp genomes. *Rosa rugosa* was used as the outgroup. Numbers at the nodes indicate bootstrap values and posterior probabilities obtained using RaxML and MrBayes, respectively. Different colors represent species belonging to different families.

To test the accuracy of the phylogenetic tree from all cp genomes, we used 53 protein-coding genes (Table S4) shared by cp genomes of 34 Caryophyllales species to construct maximum likelihood and Bayesian trees (Figure 9). The topology is generally consistent between the protein-coding genes tree with complete cp genomes tree with only slight differences in support values at some nodes; however, we also found an interesting phenomenon Amaranthaceae gathered inside Chenopodiaceae. Amaranthaceae and Chenopodiaceae are considered to be very closely related taxa. The internal relationship between Amaranthaceae and Chenopodiaceae has not been satisfactorily resolved so far. Starting from the APG II system, the Amaranthaceae and Chenopodiaceae have merged into a broad family Amaranthaceae [37]; our protein-coding gene tree supports this claim.

We selected two intergenic regions (*psaC*-*ndhE* and *psaA*-*ycf3*) with a high divergence value concatenated to construct phylogenetic trees of four *Rheum* plants and five other genera plants from the Polygonaceae family (Table S3, Fig S1). The results indicated *psaC*-*ndhE* and *psaA*-*ycf3* combinations can be used to distinguish unknown Polygonaceae plants. In the previous study, the cp *trnH*-*psbA* intergenic spacer can distinguish Polygonaceae plants [38]. Here, we proposed a new cp intergenic spacer to distinguish Polygonaceae plants.

## 3. Materials and Methods

### 3.1. Plant Materials, DNA Extraction, and Sequencing

The plant materials of *R. nobile* and *R. acuminatum* were collected from Nyingchi (Tibet, China). The specimens of *R. nobile* and *R. acuminatum* have been stored at Tibet Agriculture & Animal Husbandry University, and the specimen accession numbers are 542621150724847LY (*R. nobile*) and 542621150711217LY (*R. acuminatum*). Total genomic DNA was isolated from silica gel-dried leaf tissue using the modified CTAB method [39]. Agarose gel electrophoresis and a NanoDrop 2000 Spectrophotometer (Thermo Scientific, Carlsbad, CA, USA) were used to examine DNA integrity and concentration. Purified DNA was randomly sheared into fragments using physical methods. Paired-end sequencing libraries were constructed according to the standard Illumina protocol (Illumina, San Diego, CA, USA) and sequenced on the Illumina HiSeq X-Ten platform. The software NGS QC Toolkit v2.3.333 [40] was used to trim low-quality reads.

### 3.2. Chloroplast Genome Assembly, Annotation, and Whole-Genome Comparison

All contigs were aligned with the reference cp genome of *R. palmatum* (NC027728) via local BLAST searches [41] (http://blast.ncbi.nlm.nih.gov/). The software Geneious 11.1.4 [42] was used to assemble and annotate the cp genomes in comparison with that of *R. palmatum.* BLAST [41] and Dual Organellar GenoMe Annotator [43] (http://dogma.ccbb.utexas.edu/) were used to check the results of the annotation, which were then manually adjusted with Geneious. The circular gene maps of two *Rheum* plastomes were drawn using the Organellar Genome DRAW tool (https://chlorobox.mpimp-golm.mpg.de/OGDraw.html) [44].

Genome comparisons among the ten Polygonaceae species were performed by using mVISTA (http://genome.lbl.gov/vista/mvista/submit.shtml) [45] in Shuffle-LAGAN mode with *R. nobile* as a reference. To identify divergence hotspot regions in the four *Rheum* cp genomes, the nucleotide diversity of the *Rheum* cp genomes was evaluated using variant call format (VCF) tools [46]. Additionally, alignments of four *Rheum* cp genomes with one reference genome were carried out to detect gene rearrangements using Mauve [47]. IR expansion and contraction in the cp genomes among the seven Polygonaceae species were detected using IRscope [48] (Helsinki, Finland).

### 3.3. Characterization of Repeat Sequences and SSRs

Vmatch (http://www.vmatch.de) was used to identify the locations and lengths of repeat sequences (including forward, palindrome, reverse, and complement sequences), in the *R. nobile* and *R. acuminatum* cp genomes, with 30 bp minimum repeat size and 3 hamming distance. Simple sequence repeats (SSRs) in the cp genomes were detected using MISA [49], with the minimum numbers of repeats set to 8, 5, 3, 3, 3, and 3 for mono-, di-, tri-, tetra-, penta-, and hexanucleotides, respectively.

### 3.4. Phylogenetic Analysis

A total of 31 plastome genome sequences of Caryophyllales species were downloaded from the NCBI database (Table S3), and those of *R. nobile* and *R. acuminatum* were newly assembled in this study. *Rosa rugosa* was used as the outgroup in the phylogenetic analysis. We select 53 protein-coding genes (Table S4) shared by cp genomes of 34 species, the sequences of the complete cp genomes, and *psaC*-*ndhE* and *psaA*-*ycf3* connected together to construct maximum likelihood (ML) phylogenetic tree. In addition, 53 protein-coding genes (Table S4) concatenate to construct Bayesian trees. All gene sequence alignments were deposited into MAFFT 7.0 [50] (Osaka University, Suita, Japan), which were adjusted manually where necessary.

The ML and Bayesian trees were performed using RAxML v.8 [51] and MrBayes 3.2.6 [52, 53]. For ML analysis, the model was specified as GTRGAMMA with 1000 bootstrap replicates. The Bayesian inference tree was constructed under the GTR+I+G model (2 parallel runs, 2,000,000 generations), in which the initial 25% of sampled data were discarded as burn-in.

## 4. Conclusions

In this study, we determined and analyzed the complete cp genomes of *R. nobile* and *R. acuminatum*. Comparative analysis between *R. nobile*, *R. acuminatum*, and two other *Rheum* species was performed. The IR/SC boundary regions were relatively conserved, and only very small changes were found between these four *Rheum* cp genomes. DNA polymorphism among the four complete *Rheum* cp genomes was identified with the aim of developing molecular markers for species identification and authentication. Thirteen mutational hotspots were identified, including the *rps15*, *psbT*, *ndhF*, *matK*, *ndhH*, *petD-rpoA*, *ccsA-ndhD*, *ndhF-rpl32*, *ndhI-ndhA*, *rps16-psbK*, *psaC-ndhE*, *psaA-ycf3*, and *rpl2-psbA* regions. The phylogenetic relationships of *R. nobile* and *R. acuminatum* within Caryophyllales were consistent with the traditional morphological plant taxonomy.

## Figures and Tables

**Table 1:** Summary of the *Rheum* chloroplast genome characteristics.

Name of taxon	*R. nobile*	*R. acuminatum*	*R. palmatum*	*R. wittrockii*
GenBank accession	MK988314	MN514858	KR816224	KY985269
Genome size (bp)	161707	161655	161541	159051
LSC size (bp)	86778	86909	86518	84750
SSC size (bp)	12839	12806	13111	12999
IR size (bp)	31045	30970	30956	30651
Number of genes	131	131	131	131
Number of protein-coding genes	86	86	86	86
Number of tRNA genes	37	37	37	37
Number of rRNA genes	8	8	8	8
GC content in LSC (%)	35.3	35.4	35.4	35.4
GC content in SSC (%)	32.4	32.6	32.5	32.6
GC content in IR (%)	41.1	41.1	41.1	41.1
GC content (%)	37.3	37.4	37.3	37.5

## Data Availability

The cp genome data used to support the study findings are included in the article.

## Supplementary Materials

Supplementary Materials Table S1: list of annotated genes in *R. nobile* and *R. acuminatum* chloroplast genomes. Table S2: list of species used for interspecific comparisons. Table S3: list of species used for phylogenetic tree construction. Table S4: list of 53 protein-coding genes used for phylogenetic tree. S1 Figure: phylogenetic tree reconstruction of the 9 species inferred from maximum likelihood (ML), based on the psaC-ndhE and psaA-ycf3 genes.
