# Supplementary material for: Characterization and Comparative Analysis of Two Rheum Complete Chloroplast Genomes
Source: Biomed Res Int. 2020 Jun 19;2020:6490164. doi: 10.1155/2020/6490164 (PMC7327605; doi:10.1155/2020/6490164)
Supplement: Supplementary Materials — Table S1: list of annotated genes in R. nobile and R. acuminatum chloroplast genomes. Table S2: list of species used for interspecific comparisons. Table S3: list of species used for phylogenetic tree construction. Table S4: list of 53 protein-coding genes used for phylogenetic tree. S1 Figure: phylogenetic tree reconstruction of the 9 species inferred from maximum likelihood (ML), based on the psaC-ndhE and psaA-ycf3 genes. [file 6490164.f1.pdf]

Supporting information

Table S1. List of annotated genes in *R. nobile* and *R. acuminatum* chloroplast genomes

Groups of genes	Names of genes
Ribosomal RNAs	*rrn16*(×2), *rrn23*(×2), *rrn4.5*(×2), *rrn5*(×2)
Transfer RNAs	*trnA-UGC**(×2), *trnC-GCA, trnD-GUC, trnE-UUC, trnF-GAA, trnfM-CAU, trnG-UCC**(×2), *trnH-GUG, trnI-CAU**(×2), *trnI-GAU**(×2), *trnK-UUU*, trnL-CAA*(×2), *trnL-UAA*, trnL-UAG, trnM-CAU*(×2), *trnN-GUU*(×2), *trnP-GGG, trnP-UGG, trnQ-UUG, trnR-ACG*(×2), *trnR-UCU, trnS-GCU, trnS-GGA, trnS-UGA, trnT-GGU, trnT-UGU, trnV-GAC*(×2), *trnV-UAC*, trnW-CCA, trnY-GUA*
Proteins of small ribosomal subunit	*rps16*, rps2, rps14, rps4, rps18, rps12*(×2), *rps11, rps8, rps3, rps19, rps7*(×2), *rps15*
Proteins of large ribosomal subunit	*rpl33, rpl20, rpl36, rpl14, rpl16*, rpl22, rpl2**(×2), *rpl23*(×2), *rpl32*
Subunits of RNA polymerase	*rpoC2, rpoC1*, rpoB, rpoA*
Photosystem I	*psaB, psaA, psaI, psaJ, psaC*
Photosystem II	*psbA, psbB, psbC, psbD, psbE, psbF, psbH, psbI, psbJ, psbK, psbL, psbM, psbN, psbT, psbZ*
Cytochrome b/f complex	*petA, petB*, petD*, petG, petL, petN*
Subunits of ATP synthase	*atpA, atpB, atpE, atpF*, atpH, atpI*
Protease	*clpP* **
Large subunit of rubisco	*rbcL*
NADH dehydrogenase	*ndhA*, ndhB**(×2), *ndhC, ndhD, ndhE, ndhF, ndhG, ndhH, ndhI, ndhJ, ndhK*
Maturase	*matK*
Envelop membrane protein	*cemA*
Acetyl-CoA carboxylase	*accD*
Synthesis gene	*ccsA*
Open reading frames (ORF, ycf)	*ycf1*(×2), *ycf2*(×2), *ycf3* **, *ycf4*

* indicates a gene with one intron, and ** indicates a gene with two introns. (×2) indicates that the number of repeat units is 2.The rps12 gene is a trans-spliced gene.

Table S2. List of species used for interspecific comparisons.

	Species	GenBank Number
1	*Fagopyrum esculentum*	EU254477

2	*Fagopyrum luojishanense*	KY275182
3	*Fagopyrum tataricum*	KM201427
4	*Fallopia multiflora*	MK330002
5	*Rheum wittrockii*	KY985269
6	*Rheum palmatum*	NC027728
7	*Oxyria sinensis*	KX774248
8	*Rheum acuminatum*	MN514858
9	*Rheum nobile*	MK988314
10	*Rumex acetosa*	MH359405

Table S3 . List of species used for phylogenetic tree construction

	Species	GenBank Number	Family
1	*Silene conica*	JF715054	
2	*Silene vulgaris*	JF715057	Caryophyllaceae
3	*Pseudostellaria longipedicellata*	MH373593	
4	*Pseudostellaria okamotoi*	MH879018	
5	*Mollugo verticillata*	MK397876	Molluginaceae
6	*Trigastrotheca stricta*	MK397905	
7	*Nyctaginia capitata*	MH286318	
8	*Acleisanthes obtusa*	MH286321	Nyctaginaceae
9	*Bougainvillea spectabilis*	MK397858	
10	*Mirabilis jalapa*	MK397875	

11	*Amaranthus hypochondriacus*	KX279888	
12	*Amaranthus caudatus*	MG836508	Amaranthaceae
13	*Celosia argentea*	MK397861	
14	*Alternanthera philoxeroides*	MK795965	
15	*Drosera regia*	KY679199	Droseraceae
16	*Anredera cordifolia*	MK397854	Basellaceae
17	*Basella alba*	MK397856	
18	*Fagopyrum esculentum*	EU254477	
19	*Fagopyrum luojishanense*	KY275182	
20	*Fagopyrum tataricum*	KM201427	
21	*Fallopia multiflora*	MK330002	
22	*Rheum wittrockii*	KY985269	Polygonaceae
23	*Rheum palmatum*	NC027728	
24	*Oxyria sinensis*	KX774248	
25	*Rheum acuminatum*	MN514858	
26	*Rheum nobile*	MK988314	
27	*Haloxylon ammodendron*	KF534478	
28	*Haloxylon persicum*	KF534479	
29	*Chenopodium album*	KY419707	Chenopodiaceae
30	*Dysphania botrys*	MH898873	
31	*Dysphania pumilio*	MH936550	
32	*Chenopodium ficifolium*	MK182725	

33	*Dysphania ambrosioides*	MK182726	
34	*Rosa rugosa*	MK986659	Outgroup

Table S4 . List of 53 protein-coding genes used for phylogenetic tree construction.

	Genes		Genes		Genes
1	atpA	19	psbA	37	rpl32
2	atpB	20	psbB	38	rpl33
3	atpE	21	psbC	39	rpoA
4	atpF	22	psbD	40	rpoB
5	atpH	23	psbE	41	rpoC1
6	atpI	24	psbF	42	rpoC2
7	ccsA	25	psbH	43	rps11
8	cemA	26	psbI	44	rps12
9	clpP	27	psbJ	45	rps14
10	petA	28	psbK	46	rps16
11	petG	29	psbM	47	rps18
12	petL	30	psbN	48	rps2
13	petN	31	psbT	49	rps3
14	psaA	32	rbcL	50	rps4
15	psaB	33	rpl14	51	rps7
16	psaC	34	rpl16	52	rps8
17	psaI	35	rpl20	53	rpl32
18	psaJ	36	rpl2		

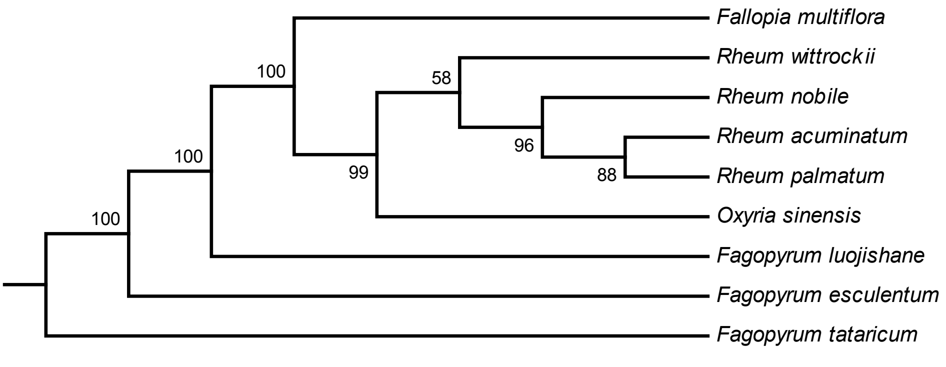

S1 Figure **Phylogenetic tree reconstruction of the 9 species inferred from maximum likelihood (ML), based on the *psaC-ndhE* and *psaA-ycf3* genes.**
